# Dissecting the Roles of Lipids in Preeclampsia

**DOI:** 10.3390/metabo12070590

**Published:** 2022-06-24

**Authors:** Yu Yang, Yixiao Wang, Yan Lv, Hongjuan Ding

**Affiliations:** Women’s Hospital of Nanjing Medical University, Nanjing Maternity and Child Health Care Hospital, Nanjing 210004, China; yangyucgz@163.com (Y.Y.); 13770982878@163.com (Y.W.)

**Keywords:** lipids, preeclampsia, pregnancy, lipidomic

## Abstract

Preeclampsia is a multisystem pregnancy disorder that is characterized by different degrees of placental malperfusion, with release of antiangiogenic factors into the circulation, leading to maternal vascular endothelial injury and high blood pressure. As a major cause of maternal and perinatal mortality and morbidity worldwide, once preeclampsia has been diagnosed, there are no curative treatments except for delivery. Lipids serve as ubiquitous and multifunctional metabolites that are integral and essential to many diverse functions on both a cellular and organismal level. Lipid metabolic abnormalities have emerged as potential risk factors for the development and progression of preeclampsia. This review comprehensively examines decades of discovery to illuminate the roles of lipids and dysregulation in the levels of various lipid classes in preeclampsia. In addition, the roles of lipids are summarized to further understand the pathogenic mechanisms of preeclampsia. Overall, the review highlights the promising potential of pathophysiology and lipid-targeting therapeutic strategies in preeclampsia.

## 1. Introduction

Preeclampsia, a disease state during pregnancy that results in high blood pressure and often kidney failure, complicates about 3–5% of all pregnancies [1,2]. Preeclampsia is well accepted as a main cause of maternal and perinatal morbidity and mortality, which is estimated to cause at least 42,000 maternal deaths annually worldwide [3]. Preeclampsia is commonly diagnosed at a routine prenatal consultation if the woman is asymptomatic but hypertensive [3].

First proposed in 1993, the two-stage paradigm of abnormal placental implantation or malperfusion followed by systemic endothelial dysfunction and severe maternal organ damage is a practical model to illustrate the pathophysiological mechanism of preeclampsia [4]. The poor early placental development drives the release of antiangiogenic molecules such as soluble fms-like tyrosine kinase-1 (sFlt1) and soluble endoglin by trophoblast cells [5,6,7], which could act on the maternal vascular endothelium to incite local endothelial release of other factors that worsen the systemic endothelium damage, including thromboxane, proinflammatory cytokines, and oxidative stress metabolites [8,9]. The pathological processes may result in acute pulmonary edema, epilepsy, liver and kidney failure, disseminated intravascular coagulation, placental abruption, fetal growth restriction, and other short-term complications during pregnancy, and they are also associated with long-term consequences following delivery, particularly cardiovascular effects and neurocognitive dysfunction [10].

Lipids are a class of water-insoluble metabolites that are generally divided into eight classes, including fatty acyls, glycerophospholipids, glycerolipids, sphingolipids, sterol lipids, prenol lipids, saccharolipids, and polyketides. They have pleiotropic roles in biology, ranging from a component of cell membranes, to cell communication, membrane trafficking, energy storage, and heat insulation [11]. Lipidomics is a subcategory of metabolomics that uses analytical chemistry techniques such as mass spectrometry and chromatography for the identification and quantification of lipids contained in biological samples [12,13,14,15]. Our understanding of the main lipid species involved in preeclampsia has substantially advanced in the past decade owing to advances in lipidomics. In this review, we will summarize recent advances that demonstrate altered lipid species involved in preeclampsia, known mechanisms that connect lipid metabolism and preeclampsia etiology, and the promising lipid-targeted therapeutics of preeclampsia.

## 2. Preeclampsia-Associated Dyslipidemia

Complex changes in lipid metabolism occur during pregnancy, probably contributing to the development of maternal dyslipidemia. Cholesterol is a common building composition of lipids and lipid-transporting particles called plasma lipoproteins [16]. Esterified cholesterol and triacylglycerols form the hydrophobic core of lipoproteins, whereas unesterified cholesterol, together with phospholipids and apolipoproteins, forms the hydrophilic surface layer.

Alteration in lipid concentration has been reported to be a risk factor for preeclampsia [17]. In addition, the accumulation of lipids and lipoproteins in the arterial wall could induce the formation of atheroma plaques and thus promote subsequent atherosclerosis [18,19]. Preeclampsia is associated with increased concentrations of triglycerides (TGs) and remnant cholesterol in early pregnancy [20]. Similarly, increased total cholesterol (TC), TGs, and low-density lipoprotein–cholesterol (LDL-C) were also documented in preeclampsia during the first trimester from a population-based prospective study, suggestive of estrogen stimulation and insulin resistance [21]. However, Hentschke et al. observed no statistical differences in maternal plasma lipid profile between preeclampsia and normal groups, probably due to only 51 samples being analyzed [22]. Further analysis showed that the ratio of TGs/high-density lipoprotein (HDL) was significantly higher in the preeclampsia group compared with the normal control women [23]. Wakabayashi et al. suggested that lipid–lipoprotein ratios have a greater value for predicting cardio-metabolic risk than isolated lipid parameters [24].

Further quantitative analysis of metabolites has been carried out to achieve accurate results despite the relationship between preeclampsia and dyslipidemia being well established. Lee et al. performed quantitative metabolite profiling of cholesterols and observed that patients with preeclampsia had significantly higher ratios of cholesterol/desmosterol and cholesterol/7-dehydrocholesterol and decreased ratios of individual cholesterol esters/cholesterol and total cholesterol esters/cholesterol when compared to women in the control group [25]. The results suggested increased cholesterol biosynthesis and increased reverse cholesterol transport, explaining that the balance in the cholesterol homeostasis regulation was disturbed in women with preeclampsia.

## 3. Lipidomics in Preeclampsia: Current Knowledge and Clinical Implications

Lipidomics approaches have been performed in several human disease studies to discover the mechanism of preeclampsia. A summary of the main studies, including their key findings, is shown in Table 1 and Table 2.

Several studies have uncovered differences in the plasma lipidomic profile of patients with preeclampsia. By conducting ultra-performance liquid chromatography-tandem mass spectrometry (UPLC-MS/MS)-based metabolomic and lipidomic profiling of placentae, Zhang et al. identified significant changes in metabolites between preeclampsia and normotensive patients, which were primarily associated with glycerophospholipids and glutathione metabolism [30]. Biomembranes are composed of lipids such as glycerophospholipids and sphingolipids that have not only a structural role but could also function as the regulator of signal transduction and immune activation pathways as well as inflammatory response [31].

He et al. demonstrated that PCs and LPCs were overall reduced in preeclampsia, which were linked to phospholipid metabolism [13]. Phospholipid metabolism is associated with preterm birth, a major clinical feature of preeclampsia [32].

Findings yielded by subsequent studies have reported glycerophospholipid species in the disease severity, and have profiled the lipidomic signature of plasma, which has been of significant value to the understanding of disease mechanisms (Table 1).

Brown et al. used shotgun lipidomics to quantify and compare the total amount and class of lipids in the placenta of preeclampsia pregnancies to explain the potential effect of placental lipid species on the development of preeclampsia [29]. The study showed that PC is the most abundant lipid in placental tissue (~36% of total lipids) from preeclampsia samples, found at 3.7 ± 1.1 μmol/g. Cholesterol was the second most abundant lipid, as the concentration was at 3.4 ± 0.8 μmol/g, representing 33% of total lipids. Moreover, a widespread higher concentration of placental neutral storage lipid content (TAG and CE) was observed in preeclampsia placentas compared to healthy controls. The key characteristics and findings of studies involved in the characterization of lipid species in the placental tissue are shown in Table 2.

## 4. Roles of Lipids in Preeclampsia Pathogenesis

**Figure 1:**
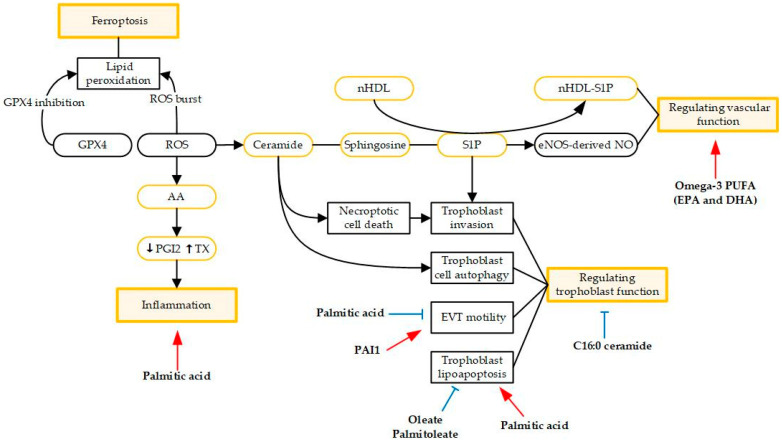
Schematic view of the roles of lipids in preeclampsia pathogenesis. Red arrows indicate stimulatory activity and bule arrows indicate inhibitory activity. Abbreviations: arachidonic acid (AA); extravillous trophoblast (EVT); high-density lipoprotein (HDL); plasminogen activator inhibitor-1 (PAI1); prostacyclin (PGI2); polyunsaturated fatty acids (PUFA); reactive oxygen species (ROS); sphingosine-1-phosphate (S1P); thromboxane (TX).

Lipid and lipid metabolism play an important role in the pathogenesis and progression of preeclampsia, including serving as regulators of vascular function and trophoblast function, driving ferroptosis, and triggering inflammatory processes (Figure 1).

### 4.1. Regulating Vascular Function

Several studies have demonstrated that altered angiogenic factors together with reduced docosahexaenoic acid (DHA) levels are associated with preeclampsia [33,34]. They also observed that maternal plasma sFlt-1 levels were higher (*p* < 0.05) in preeclampsia women and were negatively associated with DHA (*p* < 0.01). Thus, it is gradually becoming clear that lipids play an important role in angiogenesis, especially polyunsaturated fatty acids (PUFAs). Mathew et al. tested the angiogenic potential of mesenchymal stromal cells derived from the placenta, which were treated by omega-3 fatty acids. They found that supplementation with lower concentration of omega-3 fatty acids enhanced placental angiogenesis [23]. Omega-3 fatty acids have also been proven to improve the levels of vascular endothelial growth factor (VEGF) and transcription factors like PPAR-g, reducing the severity of late-onset preeclampsia rather early-onset preeclampsia [35]. In view of the lower VEGFR-1 protein levels in the early-onset preeclampsia group as compared to the control and the higher severity of angiogenic/anti-angiogenic imbalance [36], it is not surprising that maternal supplementation of omega-3 fatty acids fails to work for women with early-onset preeclampsia. Sphingolipids constitute an important class of bioactive lipids, including ceramide and sphingosine-1-phosphate (S1P). Ceramide can be deacylated by ceramidases to yield sphingosine, which can be further phosphorylated by sphingosine kinases to produce S1P. S1P could reduce vascular tone by stimulating eNOS-derived NO production [37] and thus play an important role in the regulation of vascular function [38]. Recent studies have demonstrated that S1P signaling is crucial to protecting the endothelium and maintaining vascular integrity in pregnancy [39,40]. Gaudio et al. found that sphingomyelin (SM) significantly accumulated in preeclamptic placental chorionic arteries, with impaired endothelial S1P signaling in the endothelium of these vessels [40]. Circulating neonatal HDL (nHDL) is essential to protect the feto-placental endothelial barrier since nHDL-SIP complex triggers actin filament reorganization and then enhances placental endothelial barrier function [39].

### 4.2. Driving Ferroptosis

During pregnancy, iron needs to increase substantially to support fetoplacental development and maternal adaptation to pregnancy [41]. Ferroptosis is an iron-dependent form of programmed cell death that is characterized by the accumulation of lethal lipid peroxidation, resulting in oxidative damage to cell membranes, and is recognized to differ from apoptosis, necroptosis, and autophagy in several aspects [42,43,44]. For the first time, Zhang et al. found that iron-dependent accumulation of lipid peroxides and many key proteins implicated in the regulation of ferroptosis were aberrantly expressed in the placental tissues of patients with preeclampsia [45]. GPX4 was observed to significantly decrease in preeclampsia samples whereas the expression of GPX4 had been used as an important marker for ferroptosis.

Certain lipoxygenases (LOXs) may play a prominent role in ferroptosis by oxidizing arachidonoyl and adrenoyl (AdA)—PE in the endoplasmic reticulum [46]. The PUFA diet has shown to upregulate the expression of lipoxygenase 12 (LOX12) and lipoxygenase 15 (LOX15) [47], giving a potential therapeutic strategy for preeclampsia.

### 4.3. Regulating Trophoblast Function

Despite the difference in acyl chain length, FAs differ in the number and position of unsaturation bonds because of the different number of carbons. They are commonly classified as monounsaturated (MUFA), polyunsaturated (PUFA), and saturated FAs (SFA). Palmitic acid, the most common SFA in the human body, is commonly obtained through dietary intake or synthesized endogenously from other macronutrients. Several studies have shown that palmitic acid could induce trophoblast lipoapoptosis [48] and inflammation [49]. Rampersuad et al. showed that palmitic acid restrained extravillous trophoblast (EVT) motility and induced expression of several inflammatory factors in EVTs, most notably plasminogen activator inhibitor-1 (PAI1). However, PAI1-deficient EVTs could be protected from the effects of palmitic acid on them [49]. Similarly, the supplementation of MUFAs, including palmitoleate and oleate, could also protect against trophoblast lipoapoptosis and placental injury induced by palmitic acid [48]. Therefore, the protection of palmitoleate and oleate supports the therapeutic potential of MUFAs for preeclampsia complicated with maternal obesity.

Oxidative stress status is typical of preeclampsia pathology. Melland-Smith et al. showed that the oxidative stress-induced increase in de novo synthesis led to ceramide overload, contributing to increased trophoblast cell autophagy [50]. Therefore, preeclampsia is identified as a sphingolipid-storage disorder. Liao et al. reported that preeclampsia pregnancies were associated with downregulated sphingosine kinase 1 (SPHK1) expression level [51]. Their fundings suggest a novel insight for the etiology of preeclampsia that the disrupted metabolism and signaling of S1P impair actin polymerization and YAP activation, inhibiting trophoblast invasion and thus resulting in a preeclampsia phenotype. Bailey et al. first reported that C16:0 ceramide treatment promoted necroptotic cell death in trophoblast cells particularly under conditions of caspase inhibition, thereby resulting in the placental dysfunction typical of preeclampsia [52]. Interestingly, long-chain C16:0 ceramide is also deleterious for the liver and adipose tissue, whereas very-long-chain ceramides (such as C24:0) seem to be more benign [53,54,55,56]. The C16:0 ceramides are thought to bind the mitochondrial fission factor (Mff) that initiates mitochondrial fragmentation in vitro. The interaction between sphingolipid and Mff provides a new insight for a therapeutic target for preeclampsia. Moreover, the function of very-long-chain ceramides on trophoblast cells deserves further exploration.

### 4.4. Triggering Inflammatory Processes

As one of the most important PUFAs, AA and its metabolites have attracted a lot of attention in preeclampsia, particularly in relation to inflammatory processes. Inflammation is associated with the embryo implantation process, pregnancy process, and childbirth. Although maintaining inflammation during pregnancy enables the mother to tolerate the fetus, excessive activation may result in adverse effects, such as endothelial dysfunction and maternal vascular injury [57]. Under the influence of cyclooxygenase (COX) enzymes, AA is transformed into prostacyclin and thromboxane. Increased platelet thromboxane synthesis only occurs in severe preeclampsia cases, and proinflammatory cytokines such as IL-6 and tumor necrosis factor alpha (TNF-α) are secreted excessively by maternal immune cells [58]. The imbalance in the bioactive metabolites may cause vascular stenosis and ischemia, explaining the major clinical symptoms of preeclampsia, such as hypertension, platelet aggregation, and reduced uteroplacental blood flow [58,59].

## 5. Clinical Applications of Lipids in Preeclampsia

### 5.1. Lipids as Biomarkers of Preeclampsia

Researchers have attempted to add lipid-related indicators to reach a higher detection rate since the predictive performance of using either clinical risk factors [14] or serum biomarkers [15] alone needs to be improved. He et al. investigated the potential of identifying the severity of preeclampsia patients based on lipid signatures [13]. They employed a random forest (RF) model on selected lipids, obtaining an AUC value of 0.88. According to the feature importance ranking, LPE 18:2, CER-NS d30:1, and PE 37:2 were the top three most important potential biomarkers of severe preeclampsia, with scaled feature importance scores of 0.122, 0.115, and 0.107, respectively.

A panel of mid-trimester plasma lipids and metabolites able to predict preeclampsia was also identified by Lee et al., including SM C28:1, SM C30:1, LysoPC C19:0, LysoPE C20:0, and propane-1,3-diol [12], predicting preeclampsia better than the PIGF (AUC (95% Cl): 0.868 (0.844–0.891) vs. 0.604 (0.485–0.723)) and sFlt-1/PlGF ratio. They also further validated the ability to predict preeclampsia by analyzing an at-delivery cohort, showing good discriminatory performance.

### 5.2. Lipid as a Therapeutic Target in Preeclampsia

It is notable that many of these lipid-related changes persist or worsen in later disease stages. Thus, lipid targeting could provide new therapeutic strategies for preeclampsia.

Lipoxins are identified types of endogenous anti-inflammatory lipid-based autacoids, which are the lipoxygenase-mediated biosynthesis products of AA [60]. Among them, lipoxin A4 (LXA4) and its analogues are considered a “braking signal” of inflammation, suggesting important roles in the inflammation of preeclampsia [61]. Increasing numbers of studies have demonstrated that preeclampsia is an autoimmune disease induced by pregnancy. The key feature of the disease is that it results from the autoantibody termed “angiotensin II type 1 receptor autoantibody” (AT1-AA) [62,63]. Liu et al. suggested that LXA4 suppresses AT1-AA production by modulating caspase-1 as well as enhancing phagocytosis of apoptotic trophoblast cells by macrophages, supporting caspase-1 serving as a therapeutic target for attenuating AT1-AA and LXA4 to protect patients from preeclampsia [64].

Statins have been proposed as a highly promising candidate for the prevention and treatment of preeclampsia. Their primary target is 3-hydroxy-3-methylglutaryl-coenzyme (HMG-CoA), the rate-limiting enzyme in cholesterol biosynthesis [10]. Statins are thought to improve endothelial function and reduce circulating inflammatory cytokines by up-regulating antioxidant pathways, inhibiting transcription factors that promote inflammation, and impairing the immune response of T-helper cells [65]. Data from animal models of preeclampsia demonstrated that pravastatin could reduce circulation sFlt-1 concentrations, prevent vascular dysfunction, and improve cardiac output postpartum [66,67]. Preliminary human data have confirmed the overall safety and favorable pregnancy outcomes of pravastatin in women at high risk for preeclampsia [68]. However, DöBert et al. conducted a multicenter, double-blind, placebo-controlled trial of 1120 women at high risk of prenatal preeclampsia and then evaluated the effects of pravastatin at 20 mg daily or placebo. Surprisingly, they concluded that pravastatin has no benefit in reducing the incidence of preeclampsia [69]. It is possible that considerably higher doses and earlier duration of treatment with pravastatin are needed to prevent the development of preeclampsia.

## 6. Conclusions and Future Perspectives

Clinical and basic studies from the last decade have clearly demonstrated that lipids play an important role in the pathogenesis and progression of preeclampsia. Although highly promising, further exploration of the underlying mechanisms remains a challenge considering the lack of a fully deciphered placental lipidome due to current technical limitations. Moreover, available animal models that can emulate metabolic features of human preeclampsia are needed for further investigation. More clinical trials should be planned to definitively confirm whether pravastatin can benefit patients complicated with preeclampsia.

The presented findings strongly suggest that lipid metabolism is essential to the development of preeclampsia, and novel associations drawn from it have the potential to open new research opportunities. Future studies should aim at elucidating the identification of more accurate biomarkers and at the development of effective, safe, and personalized drugs targeting key steps in lipid metabolism for the treatment of preeclampsia.

## Figures and Tables

**Table 1:** Summary of studies on lipid profiling of plasma or serum samples of patients with preeclampsia. (“↑” represents an increase in the level of the metabolite; “↓” represents a decrease in the level of the metabolite).

Author and Reference	Type of Study	Population Features Sample Size	Key Finding/s	Analytical Approach
Anand 2016 [26]	Nested case-control study	Serum samples all collected at 12–14 weeks. A discovery study. Later developed pre-eclampsia (*n* = 29); controls with uncomplicated pregnancies (*n* = 27). A second confirmatory study. Later developed pre-eclampsia (*n* = 37); controls with uncomplicated pregnancies (*n* = 43).	↑Glycerophosphocholines, oxPC; 45 potential pre-eclampsia markers were detected and 23 markers continued to be statistically significant, suggesting that measurement of serum lipid biomarkers early in pregnancy would identify patients at risk for subsequent development of preeclampsia.	TOF LC/MS
Odenkirk 2020 [27]	Case-control study	Plasma samples of pregnant women admitted to labor and delivery. Term pre-eclampsia (*n* = 48); healthy normal controls (*n* = 98).	↑DGs, TG 18:0, LysoPCs (except LysoPC 0:0_14:0) ↓PI, PCs, TG 18:3 in women with preeclampsia compared with healthy pregnant women.	LC-IMS-MS
Lee 2020 [12]	Case-control study; retrospective cohort study	Plasma samples collected at 16–24 weeks. Later developed pre-eclampsia (*n* = 33); normal controls (*n* = 66). Plasma samples collected within 3 days of delivery from pre-eclampsia patients (*n* = 13) and normal controls (*n* = 21).	A mid-trimester biomarker panel for the prediction of preeclampsia with five metabolites (SM C28:1, SM C30:1, LysoPC C19:0, LysoPE C20:0, propane-1,3-diol), predicting preeclampsia better than PIGF (AUC (95%CI): 0.868 (0.844–0.891) vs. 0.604 (0.485–0.723)) and sFlt-1/PIGF ratio.	GC-TOF-MS LC-Orbitrap MS
He 2021 [13]	Case-control study	Plasma samples from RMATRIX Hawaii Biorepository. Severe pre-eclampsia (*n* = 44); healthy pregnant women (*n* = 20).	↑PE 37:2, OxPE, OxPC ↓Cer (Cer-NS d30:1), PCs, LPCs. Various PCs and LysoPCs mediate severe preeclampsia through PC 35:1e.	LC-MS/MS

**Table 2:** Summary of studies on lipid profiling of placental tissue samples of patients with preeclampsia. (“↑” represents an increase in the level of the metabolite; “↓” represents a decrease in the level of the metabolite).

Author and Reference	Type of Study	Population Features Sample Size	Key Finding/s	Analytical Approach
Austdal 2015 [28]	Case-control study	Placenta samples of preeclampsia pregnant women (*n* = 19) and normotensive pregnant women (*n* = 15) who delivered by cesarean section.	Glycerophosphocholines↑ Placentas from preeclamptic pregnancies showed enrichment of phospholipid biosynthesis compared to placentas from normotensive pregnancies.	HR-MAS MRS
Brown 2016 [29]	Case-control study	Placental lipid profiles from preeclampsia pregnancies (*n* = 23) were compared to healthy pregnancies (*n* = 68).	↑TAG, CE, Cholesterol, PC 18:0_22:6, were higher in placenta complicated with preeclampsia compared to healthy controls.	QTRAP-MS
Zhang 2022 [30]	Case-control study	Placenta samples of preeclampsia pregnant women (*n* = 12) and normotensive pregnant women (*n* = 14) who delivered by cesarean section.	↑PG 38:5, PG 40:5, ↓PC 36:4e, PE 40:7; Glutathione metabolism and glycerophospholipid metabolism were closely related to preeclampsia.	UPLC-MS/MS

## Abbreviations

AUC, area under the curve; CE, cholesteryl ester; CER, ceramide; DG, diacylglycerol; GC-TOF MS, gas chromatography time-of-flight mass spectrometry; HR-MAS MRS, high-resolution magic angle spinning nuclear magnetic resonance spectroscopy; LC-Orbitrap MS, liquid chromatography Orbitrap mass spectrometry; Ox, oxidized; PC, phosphatidylcholine; PE, phosphatidylethanolamine; PG, phosphatidylglycerol; PI, phosphatidylinositol; QTRAP-MS, trap-triple quadrupole mass spectrometer; SM, sphingomyelin; TOF, time-of-flight mass spectrometry; UHPLC, ultra-high-performance liquid chromatography; UPLC, ultra-performance liquid chromatography.
